# Supplementary material for: Synthetic recovery of impulse propagation in myocardial infarction via silicon carbide semiconductive nanowires
Source: Nat Commun. 2022 Jan 10;13:6. doi: 10.1038/s41467-021-27637-2 (PMC8748722; doi:10.1038/s41467-021-27637-2)
Supplement: Supplementary file 1 — Supplementary Information [file 41467_2021_27637_MOESM1_ESM.pdf]

Synthetic recovery of impulse propagation in myocardial infarction via silicon carbide semiconductive nanowires.

Paola Lagonegro[1,2#], Stefano Rossi[3#], Nicolò Salvarani[4,5], Francesco Paolo lo Muzio[3,6], Giacomo Rozzi[3,4], Jessica Modica[4,5], Franca Bigi[7,1], Martina Quaretti[7,1], Giancarlo Salviati[1], Silvana Pinelli[3], Rossella Alinovi[3], Daniele Catalucci[4,5], Francesca D'Autilia[4], Ferdinando Gazza[8], Gianluigi Condorelli[4,9], Francesca Rossi[1§] and Michele Miragoli[3,4§*].

1. Istituto dei Materiali per l'Elettronica e il Magnetismo (IMEM), National Research Council CNR, Parco Area delle Scienze 37/A 43124 Parma, IT
2. Istituto di Scienze e Tecnologie Chimiche "Giulio Natta", Consiglio Nazionale delle Ricerche (SCITEC-CNR), Via A. Corti 12, 20133, Milan, IT
3. CERT Centro di Eccellenza per la Ricerca Tossicologica, Dipartimento di Medicina e Chirurgia Università di Parma, Via Gramsci 14, 43124 Parma, IT
4. Humanitas Research Hospital - IRCCS, Via Manzoni 56, 20089 Rozzano (Milan), IT
5. Istituto di Ricerca Genetica Biomedica (IRGB), National Research Council CNR, UOS Milan, Via Fantoli 16/15, 20138 Milan, IT
6. Dipartimento di Scienze Chirurgiche Odontostomatologiche e Materno-Infantili, Università di Verona, Policlinico G.B. Rossi, - P.le L.a. Scuro 10, 37134 Verona, IT
7. Dipartimento di Scienze Chimiche, della Vita e della Sostenibilità Ambientale, Università di Parma, Parco Area delle Scienze, 11/a - 43124 Parma, IT
8. Dipartimento di Scienze Medico-Veterinarie, Università di Parma, via del Taglio 10, 43126 Parma, IT
9. Humanitas University, Via Rita Levi Montalcini 4, 20090 Pieve Emanuele (Milan), IT

# These authors contributed equally

§ These authors jointly supervised this work

* Corresponding author:

Michele Miragoli

Dipartimento di Medicina e Chirurgia,

Università di Parma, via Gramsci 14, 43124 Parma (IT)

Tel: +39 0521933256

Email: michele.miragoli@unipr.it

Supplemental Materials

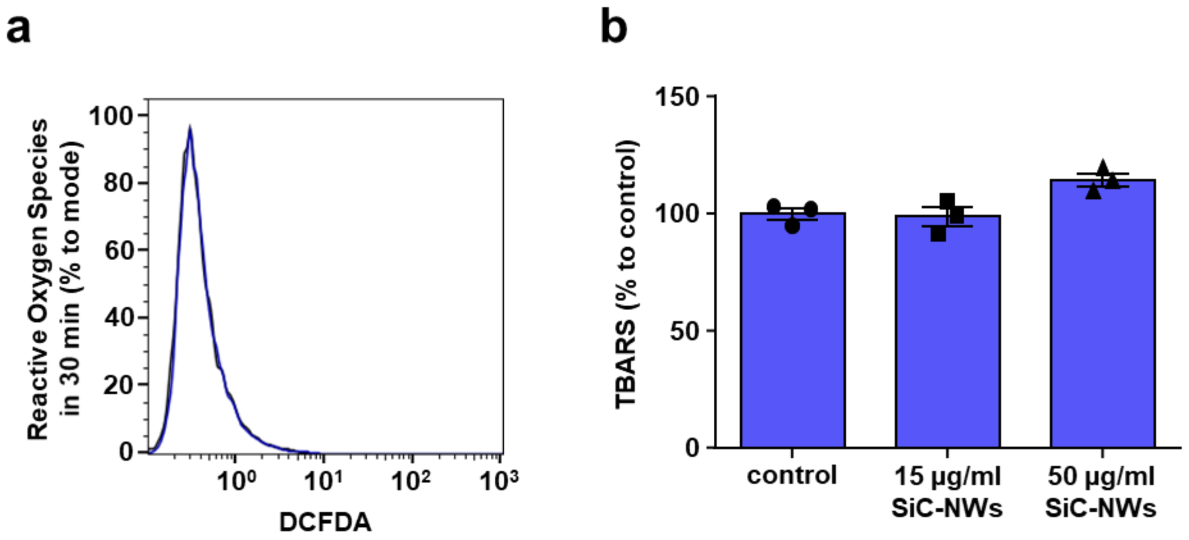

Supplementary Figure 1. Reactive oxygen species and ROS-induced lipid peroxidation in cardiomyocytes. a. ROS production after 30 min in the presence of SiC-NWs. (black line: control; blue line: 50 µg/ml SiC-NWs). DCFDA: Dichlorofluorescin Diacetate (n=3 experiments repeated with similar results). **b**. Thiobarbituric acid reactive substances (TBARS) produced by ROS-induced lipid peroxidation (n=3 experiments for each gruop). Data expressed as % of Control (mean ± S.E.M.). $p=0.90$ for 15 µg SiC-NWs vs Control; $p=0.10$ for 50 µg SiC-NWs vs Control. Mann-Whitney U test. Statistical significance set at $p<0.05$. Source data are provided as a Source Data file.

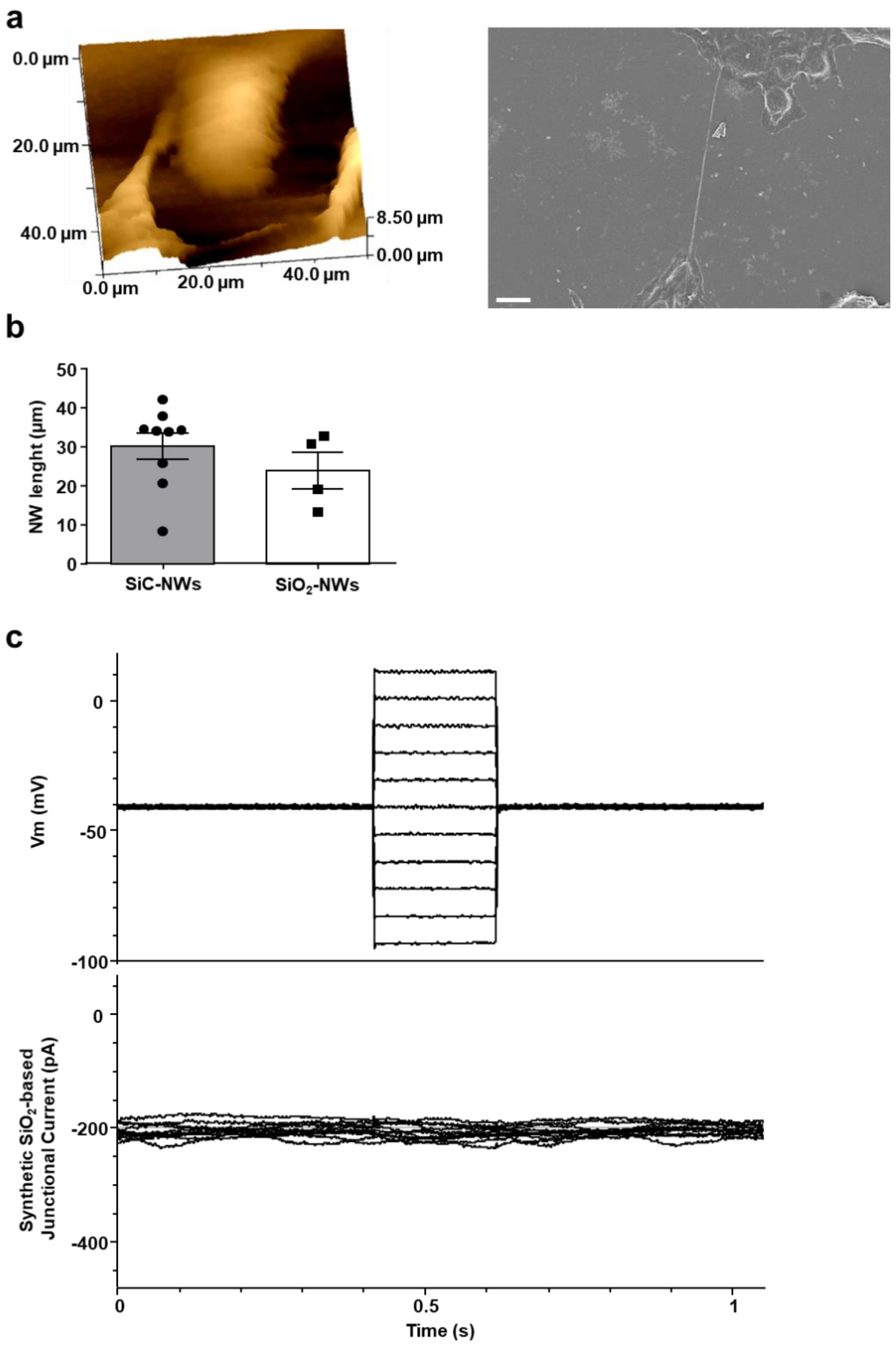

Supplementary Figure 2. MNB formation and uncoupling evidence for SiO_2-NWs synthetically connected cardiomyocytes. a. Hopping probe SICM (left) and SEM (right) images showing MNB formation (Scale bar: 10µm). N=4 experiments repeated with similar results. **b**. Average nanowire length obtained by SICM (maximal XY range 100x100 µm) acquired for SiO_2-NWs (n=4 experiments repeated with similar results) and SiC-NWs (n=9 experiments repeated with similar results) showing no difference in the MNB formation and dimension. Data are presented as mean

± S.E.M. Unpaired two-side Student's t-test. p=0.32. Statistical significance set at $p<0.05$. **c**. Vm protocol (top) and absence of junctional coupling (bottom) for SiO_2-NWs (n=5 experiments repeated with similar results). Source data are provided as a Source Data file.

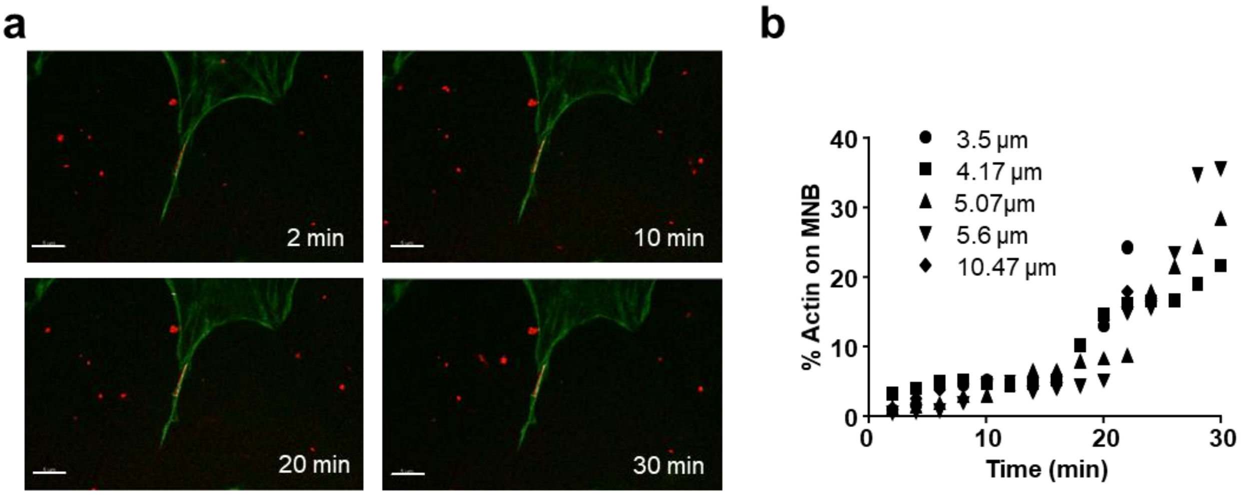

Supplementary Figure 3. Biodynamic interface in partial internalization of SiC-NWs in cardiac myofibroblasts. **a.** Time-lapse recording of the biodynamic interface in neonatal rat ventricular myofibroblasts live-stained with actin (green) and SiC-NW (red) over 30 min. Scale bars: 5µm. N=10 experiments repeated with similar results. **b**. Membrane nanobridge formation triggered by actin for SiC-NWs internalization from myofibroblasts (MFB, n=5). Numbers indicate SiC-NW lengths. Source data are provided as a Source Data file.

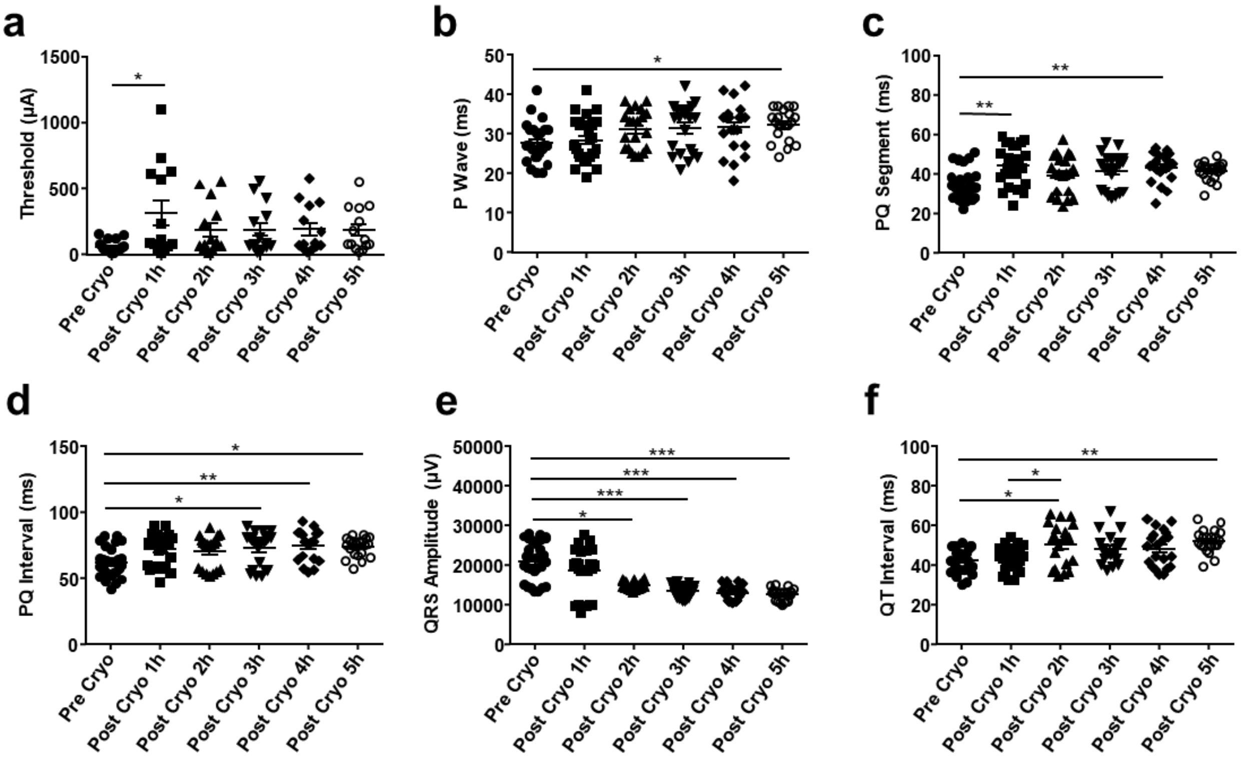

Supplementary Figure 4. Stabilization of EGs after cryoinjury. EGs parameters for (**a**)Threshold (Pre Cryo: n=19; Post Cryo 1h: n=14; Post Cryo 2h: n=14, Post Cryo 3h: n=14, Post Cryo 4h: n=14; Post Cryo 5h: n=14), (**b**) P wave duration (Pre Cryo: n=28; Post Cryo 1h: n=28; Post Cryo 2h: n=21, Post Cryo 3h: n=21, Post Cryo 4h: n=21; Post Cryo 5h: n=21), (**c**) PQ segment (Pre Cryo: n=28; Post Cryo 1h: n=24; Post Cryo 2h: n=21, Post Cryo 3h: n=21, Post Cryo 4h: n=21; Post Cryo 5h: n=21), (**d**) PQ interval (Pre Cryo: n=28; Post Cryo 1h: n=24; Post Cryo 2h: n=21, Post Cryo 3h: n=21, Post Cryo 4h: n=21; Post Cryo 5h: n=21), (**e**) QRS amplitude (Pre Cryo: n=28; Post Cryo 1h: n=27; Post Cryo 2h: n=21, Post Cryo 3h: n=21, Post Cryo 4h: n=21; Post Cryo 5h: n=21)and (**f**) QT interval (Pre Cryo: n=27; Post Cryo 1h: n=27; Post Cryo 2h: n=21, Post Cryo 3h: n=21, Post Cryo 4h: n=21; Post Cryo 5h: n=21) before (Pre Cryo) and in the 5 hours following the ventricular cryoinjury. Data are presented as Mean ± S.E.M. Threshold, P Wave, PQ segment, PQ interval, QRS amplitude: Kruskal-Wallis (post hoc analyses: Dunn's multiple comparison. C.I.=95%). QT interval: Ordinary one-way ANOVA (post hoc analyses: Tukey's multiple comparison. C.I.=95%). *p<0.05, **p<0.01, ***p<0.001. Source data are provided as a Source Data file.

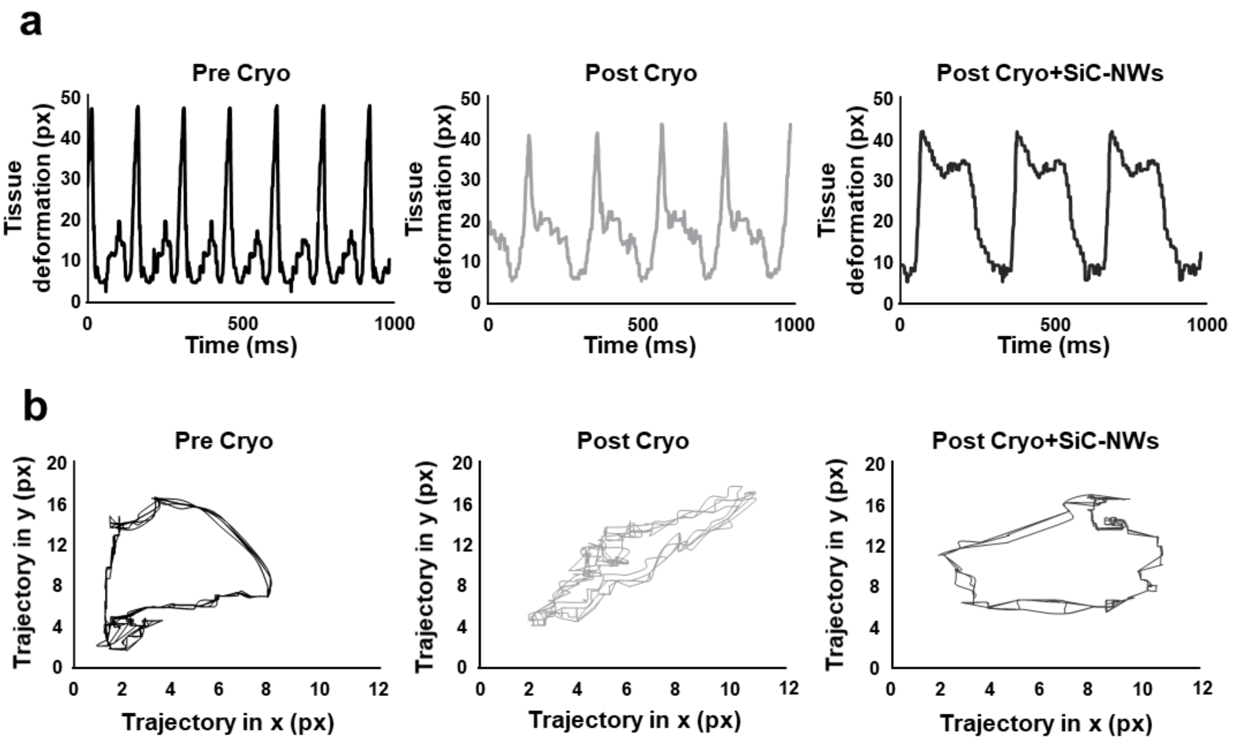

Supplementary Figure 5. Video kinematic evaluation of epicardial motion. a. Tissue deformation traces for Pre Cryo, Post Cryo and Post Cryo+ SiC-NWs groups during cardiac cycles in 1 s recording. **b**. Same as (a) for x and y trajectories. N=5 experiments repeated with similar results.

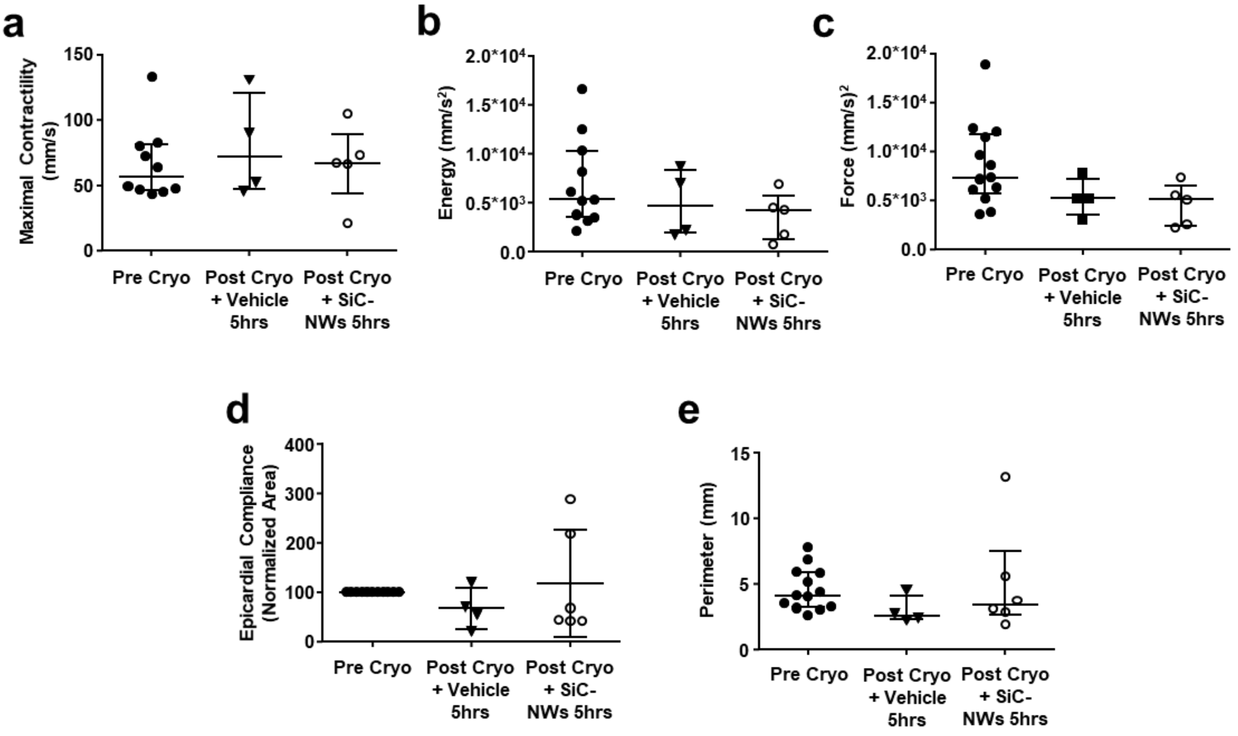

Supplementary Figure 6. Video Kinematic Evaluation of Epicardial movement during the cardiac cycle. Epicardial kinematics at the control point (Pre Cryo, black dots, n=13), 5 h after Vehicle injection in the cryoinjury (black triangles, n=4), and 5 h after the injection of 1 mg of SiC-NWs (open circles, n=6), for contractility (**a**), energy (**b**), and force (**c**). **d-e**. Same as a for trajectory area and trajectory perimeter, as indexes of ventricular compliance, respectively. Kruskal-Wallis (*post hoc* analyses: Dunn's multiple comparison). Data are represented as median and interquartile range. Statistical significance set at $p<0.05$. Source data are provided as a Source Data file.
