# Supplementary material for: Synthetic recovery of impulse propagation in myocardial infarction via silicon carbide semiconductive nanowires
Source: Nat Commun. 2022 Jan 10;13:6. doi: 10.1038/s41467-021-27637-2 (PMC8748722; doi:10.1038/s41467-021-27637-2)
Supplement: Supplementary file 3 — Description of Additional Supplementary Files [file 41467_2021_27637_MOESM3_ESM.docx]

**Description of Additional Supplementary Files**

**File Name:** Supplementary Video 1

**Description:** Time-lapse imaging recording of MNB formation by actin on red-labeled SiC-NW in HL1 cells.

**File Name:** Supplementary Video 2

**Description:** Time-lapse imaging recording of MNB formation by actin on red-labeled SiC-NW in cardiac myofibroblasts.

**File Name:** Supplementary Video 3

**Description:** Synchronized intracellular Ca2+ activity in synthetically SiC-NWs coupled HL1 cells.
