# Supplementary material for: Synthetic recovery of impulse propagation in myocardial infarction via silicon carbide semiconductive nanowires
Source: Nat Commun. 2022 Jan 10;13:6. doi: 10.1038/s41467-021-27637-2 (PMC8748722; doi:10.1038/s41467-021-27637-2)
Supplement: Supplementary file 7 — Reporting Summary [file 41467_2021_27637_MOESM7_ESM.pdf]

nature portfolio

Corresponding author(s): Michele Miragoli

Last updated by author(s): Nov 12, 2021

Reporting Summary

Nature Portfolio wishes to improve the reproducibility of the work that we publish. This form provides structure for consistency and transparency in reporting. For further information on Nature Portfolio policies, see our Editorial Policies and the Editorial Policy Checklist.

Statistics

For all statistical analyses, confirm that the following items are present in the figure legend, table legend, main text, or Methods section.

n/a	Confirmed	
[ ]	[x]	The exact sample size (n) for each experimental group/condition, given as a discrete number and unit of measurement
[ ]	[x]	A statement on whether measurements were taken from distinct samples or whether the same sample was measured repeatedly
[ ]	[x]	The statistical test(s) used AND whether they are one- or two-sided<br>*Only common tests should be described solely by name; describe more complex techniques in the Methods section.*
[x]	[ ]	A description of all covariates tested
[ ]	[x]	A description of any assumptions or corrections, such as tests of normality and adjustment for multiple comparisons
[ ]	[x]	A full description of the statistical parameters including central tendency (e.g. means) or other basic estimates (e.g. regression coefficient) AND variation (e.g. standard deviation) or associated estimates of uncertainty (e.g. confidence intervals)
[ ]	[x]	For null hypothesis testing, the test statistic (e.g. F, t, r) with confidence intervals, effect sizes, degrees of freedom and P value noted<br>*Give P values as exact values whenever suitable.*
[x]	[ ]	For Bayesian analysis, information on the choice of priors and Markov chain Monte Carlo settings
[x]	[ ]	For hierarchical and complex designs, identification of the appropriate level for tests and full reporting of outcomes
[x]	[ ]	Estimates of effect sizes (e.g. Cohen's d, Pearson's r), indicating how they were calculated

Our web collection on statistics for biologists contains articles on many of the points above.

Software and code

Policy information about availability of computer code

Data collection	Commercial Software: IonScope Ltd (UK); pClamp 10.6 (USA): MiCAM Ultima acquisition Software (USA), Crescent Electronics (USA)
Data analysis	Commercial Software: FlowJo software package (Tree Star Inc, OR, USA) ; IonScope Ltd (UK): ImageJ- Fiji 1.51a (USA); ClampFit 10.6 (USA); BrainVision 10.2; Graphad 6.0 (Prism, USA), Imaris 7.4.2 (Bitplane, CH), ViKiE kinematic analysis software (Matlab, Mahworks, IT)

For manuscripts utilizing custom algorithms or software that are central to the research but not yet described in published literature, software must be made available to editors and reviewers. We strongly encourage code deposition in a community repository (e.g. GitHub). See the Nature Portfolio guidelines for submitting code & software for further information.

Data

Policy information about availability of data

All manuscripts must include a data availability statement. This statement should provide the following information, where applicable:

- Accession codes, unique identifiers, or web links for publicly available datasets
- A description of any restrictions on data availability
- For clinical datasets or third party data, please ensure that the statement adheres to our policy

The authors declare that a complete data availability statement that supporting the findings of this study are available within the paper [and its supplementary information files]

Field-specific reporting

Please select the one below that is the best fit for your research. If you are not sure, read the appropriate sections before making your selection.

☒ Life sciences ☐ Behavioural & social sciences ☐ Ecological, evolutionary & environmental sciences

For a reference copy of the document with all sections, see nature.com/documents/nr-reporting-summary-flat.pdf

Life sciences study design

All studies must disclose on these points even when the disclosure is negative.

Sample size	In accordance with the 3R's, studies were designed to reduce the number of animals used.<br>The size of each group was established a priori accordingly to statistical criteria on the basis of the results of the Power analysis (G * Power Version 3.1.2; Franz Faul, Universita Kiel, Germany) predicting an error alpha = 0.05, 1-beta = 0.80 . From retrospective data (preliminary and scientific publications), we predict an R2 value of 0.8 for experiments corresponding to N = 6, alpha = 0.05, at 80% power, assuming a linear regression with 2 main covariates. Therefore by association 6±2 rats per group will be used to maximize statistical power.<br>For Cryo and Vehicle groups (that we considered as control groups) our previous works demonstrated that a number of 4 is sufficient to reach statistical significance. For SiC-NWs group we selected a number of 6 taking in consideration the violation of normality assumption.
Data exclusions	Outlier data have been detected via GraphPad 6.0 and excluded from the final analysis. The exclusion criteria were not pre-established.
Replication	All measurements present in this work were performed independently
Randomization	In-vivo experiments were randomized chronologically in terms of the acute treatment as follow: 3 animals SiC-NWs, 1 animal Vehicle; 1 animal SiC-NWS, 4 animal sCryo, 3 animals Vehicle and 2 animals SiC-NWs. In-vitro experiment the samples were allocated randomly in the experimental groups.
Blinding	For the in-vitro experiments, patch-clamp and optical mapping and toxicological analysis has been performed and analyzed "single-blind", in terms that operators were not aware if the culture containing SiC-NWs or SiO2-NWs during analysis. For the in-vivo experiments all the investigators were also blinded to group allocation during data collection and analysis .

Reporting for specific materials, systems and methods

We require information from authors about some types of materials, experimental systems and methods used in many studies. Here, indicate whether each material, system or method listed is relevant to your study. If you are not sure if a list item applies to your research, read the appropriate section before selecting a response.

Materials & experimental systems

n/a	Involved in the study	
☒	☐	Antibodies
☐	☒	Eukaryotic cell lines
☒	☐	Palaeontology and archaeology
☐	☒	Animals and other organisms
☒	☐	Human research participants
☒	☐	Clinical data
☒	☐	Dual use research of concern

Methods

n/a	Involved in the study	
☒	☐	ChIP-seq
☐	☒	Flow cytometry
☒	☐	MRI-based neuroimaging

Eukaryotic cell lines

Policy information about cell lines

Cell line source(s)	HL1 cardiac muscle cell line (Merck, IT)
Authentication	no authentication procedure
Mycoplasma contamination	cell line tested negative for mycoplasma contamination
Commonly misidentified lines<br>(See ICLAC register)	no commonly misidentified cell lines were used in the study

Animals and other organisms

Policy information about studies involving animals; ARRIVE guidelines recommended for reporting animal research

Laboratory animals	14 Sprague Dawley rats (both sexes) (8-10 months old, weighting 300-350 g) bred in our animal facility approved protocols: PMS53/2009, 281/2017, 989/2017.<br>10 neonatal Sprague Dawley 1 days old rats for myofibroblasts isolation and culture. D2326/2019
Wild animals	the study did not involved wild animals
Field-collected samples	Animals were bred in our animal facility, maintaned singly housed with the light on between 7 a.m. and 7 p.m. in a temperature-controlled room at 22–24 °C. The bedding of the cages consisted of wood shavings with food and water available ad libitum. No field collected samples were used in this study
Ethics oversight	University of Parma, Animal Welfare (Organismo Preposto per il Benessere Animale, OPBA); Ministry of Health D.L.4/3/2014, 2010/63/UE

Note that full information on the approval of the study protocol must also be provided in the manuscript.

Flow Cytometry

Plots

Confirm that:

- [x] The axis labels state the marker and fluorochrome used (e.g. CD4-FITC).
- [x] The axis scales are clearly visible. Include numbers along axes only for bottom left plot of group (a 'group' is an analysis of identical markers).
- [x] All plots are contour plots with outliers or pseudocolor plots.
- [x] A numerical value for number of cells or percentage (with statistics) is provided.

Methodology

Sample preparation	The cell phase distribution was determined by DNA content, as already described. Briefly, cells were fixed in ethanol, stained with propidium iodide, and then sorted in cytofluorimeter. Intracellular ROS generation was investigated employing 2′,7′-dichlorodihydrofluorescein diacetate (DCFH-DA). Hydrogen peroxide (50 µmol/l) was adopted as a positive control.
Instrument	FC500™ flow cytometer
Software	FlowJo software package (Tree Star Inc., OR, USA).
Cell population abundance	We used a single HL1-cell line and acquired 20.000 events in all experiments.
Gating strategy	Doublet discrimination and exclusion has been performed for the cell cycle analysis. No gating strategy has been used in the FACS data

- [ ] Tick this box to confirm that a figure exemplifying the gating strategy is provided in the Supplementary Information.
